# Editorial: Signaling Events in Regulating Leaf Senescence

**DOI:** 10.3389/fpls.2022.860923

**Published:** 2022-02-23

**Authors:** Yongfeng Guo, Salma Balazadeh, Nam-Chon Paek

**Affiliations:** ^1^Tobacco Research Institute, Chinese Academy of Agricultural Sciences (CAAS), Qingdao, China; ^2^Institute of Biology, Leiden University, Leiden, Netherlands; ^3^Department of Agriculture, Forestry and Bioresources, Seoul National University, Seoul, South Korea

**Keywords:** leaf senescence, signals, transcription factor, senescence-associated genes (SAGs), jasmonic acid (JA)

Leaf senescence is a critical stage in plant life cycles and is of great importance in agriculture (Woo et al., 2019; Guo et al., 2021). Initiation and progression of leaf senescence occur under the finely-tuned control of a complex network of signaling events that can be triggered by a variety of signals and environmental cues. Senescence-regulating signals, including age, reproductive growth, phytohormones, abiotic/biotic stresses, and small peptides, as reported recently (Zhang et al., 2021), are often perceived by membrane-localized receptors and transduced into the cells to trigger differential expression of thousands of genes, especially senescence-associated genes (SAGs), many of which function in regulating leaf senescence (Ahmad and Guo, 2019). During the past two decades, a significant number of genes that are involved in senescence regulation have been characterized (Woo et al., 2019; Guo et al., 2021), including transcription factors that are potentially responsible for regulating the massive switch in gene expression during leaf senescence (Kim et al., 2016; Li et al., 2018; Li et al.). The big picture of regulatory networks of leaf senescence, however, remains to be unraveled.

Three of the articles included in this Research Topic are related to senescence- regulating signals. Research progress on senescence regulation by light and circadian clock was summarized in a mini-review by Lee et al.. Involvement of Phytochrome-Interacting Factors (PIFs) from light signaling and core clock components in senescence processes suggested important roles of light as senescence-suppressing and circadian clock as senescence-inducing signals (Lee et al.). ABA has been shown to be a senescence-promoting signal in a number of plant species (Guo et al., 2021). A sharp increase in abscisic acid (ABA) content was detected during winter in senescing leaves and in rhizomes of yellow flag (*Iris pseudacorus*) plants growing in a natural wetland, suggesting a major role of ABA in regulating cold-induced leaf senescence in this wetland plant (Caselles et al.). In studying phytotoxic effects of tropospheric ozone (O_3_) on the foliage of hybrid poplar, Turc et al. found that precocious senescence and hypersensitive response-like lesions were induced on leaves after O_3_ exposure. Higher O_3_ tolerance was observed in younger leaves than older leaves (Turc et al.), confirming the role of O_3_ as a senescence-promoting signal.

As critical regulators of gene expression change during leaf senescence, a large number of transcription factors have been characterized to be involved in senescence regulation (Guo, 2013; Woo et al., 2019). In this collection of articles, one NAC and two WRKY transcription factors were studied for their regulatory roles in leaf senescence (Kan et al.; Li et al.; Qiao et al.). The Arabidopsis NAC075 transcription factor was found to function as a negative regulator of leaf senescence. Loss-of-function promoted, while overexpression of *NAC075* delayed senescence of Arabidopsis leaves. Further study suggested that NAC075 directly suppresses the expression of the antioxidant enzyme gene *CAT2*, thereby promoting the accumulation of reactive oxygen species (ROS) to control leaf senescence (Kan et al.). Similarly, the rice WRKY transcription factor OsWRKY93 was identified as a negative regulator of dark-induced leaf senescence and susceptibility to *Magnaporthe oryzae* infection. CRISPR/Cas9-edited mutants of *OsWRKY93* showed early senescence and higher disease sensitivity while enhanced expression of this gene led to delayed senescence and resistance to *M. oryzae* infection (Li et al.). The wheat WRKY family protein, TaWRKY13-A, on the other hand, acted as a positive regulator of leaf senescence (Qiao et al.). VIGS-silencing of *TaWRKY13-A* led to delayed senescence in leaves whereas overexpression of this gene accelerated the onset of leaf senescence. Moreover, the function of TaWRKY13-A in regulating leaf senescence seemed to be related to the jasmonic acid (JA) signaling pathway (Qiao et al.).

Transcription factors often function in activating the expression of *SAG*s, which leads to the execution of senescence via various biochemical and physiological processes (Guo, 2013; Woo et al., 2019). Some of the SAGs might be involved in protein degradation, such as the Ring/U-box protein AtUSR1, which was shown to be involved in age-dependent and dark-induced leaf senescence in Arabidopsis (Zhang et al.). AtUSR1 was identified as a positive regulator of senescence that functions downstream of the MYC2-mediated JA signaling pathway. MeJA treatment promoted *AtUSR1* expression in a MYC2-dependent manner. While the *myc2* mutation alone caused a delay in leaf senescence, overexpression of *AtUSR1* in the *myc2* background led to precocious senescence (Zhang et al.). Some other senescence-regulating genes encode for catalytic enzymes in various metabolic and biochemical processes. Functional inactivation of UDP-N-acetylglucosamine pyrophosphorylase 1 (UAP1) induced defense-related lesion-mimic spots and early senescence in rice leaves. UAP2 showed similar catalytic activities as UAP1 and overexpression of *UAP2* rescued the *uap1* mutant phenotype. It was suggested that UAP1 and UAP2 play key roles in rice leaf senescence in a synergetic manner (Wang et al.). Another rice gene, *CYP71P1*, was identified via map-based cloning of the causal gene of two lesion mimic mutants (*msl-1* and *msl-2*) obtained from ethyl methyl sulfonate mutagenesis. CYP71P1 is a cytochrome P450 monooxygenase and was shown to be involved in the regulation of leaf senescence and cell death (Zheng et al.). Also identified via map-based cloning, ACCELERATED CELL DEATH 6 (ACD6) is a transmembrane ankyrin repeat protein functioning in sequential and monocarpic senescence in Arabidopsis (Jasinski et al.). The results of ^15^N partitioning experiments showed that N remobilization efficiency was significantly lower in the *acd6* mutant than the wild type. ACD6 did not affect nitrate uptake efficiency but enhanced nitrogen remobilization to seeds (Jasinski et al.).

Interestingly, most of the senescence regulators described in this Research Topic are also involved in stress responses. OsWRKY93, ACD6, UAP1, and UAP2 are involved in disease resistance (Li et al.; Jasinski et al.; Wang et al.). NAC075, OsWRKY93, AtUSR1, and CYP71P1 are regulators of ROS homeostasis (Kan et al.; Li et al.; Zhang et al.; Zheng et al.). Both AtUSR1 and TaWRKY13-A function through the JA signaling pathway (Zhang et al.; Qiao et al.), which is related to biotic and abiotic stress responses (Wang et al., 2021). All these results indicate extensive cross talk between leaf senescence and stress responses.

## Author Contributions

All authors contributed to this manuscript and approved the final version.

## Funding

YG was supported by the Agricultural Science and Technology Innovation Program of China, Chinese Academy of Agricultural Sciences (ASTIP-TRI02), and Funds for Special Projects of the Central Government in Guidance of Local Science and Technology Development (21-1-1-1-zyyd-nsh).

## Conflict of Interest

The authors declare that the research was conducted in the absence of any commercial or financial relationships that could be construed as a potential conflict of interest.

## Publisher's Note

All claims expressed in this article are solely those of the authors and do not necessarily represent those of their affiliated organizations, or those of the publisher, the editors and the reviewers. Any product that may be evaluated in this article, or claim that may be made by its manufacturer, is not guaranteed or endorsed by the publisher.
